# Differential contributions of two domains of NAI2 to the formation of the endoplasmic reticulum body

**DOI:** 10.3389/fpls.2023.1184678

**Published:** 2023-06-06

**Authors:** Yun Ju Choi, Kyoung Rok Geem, Jitae Kim, Dong Wook Lee

**Affiliations:** ^1^ Department of Integrative Food, Bioscience and Biotechnology, Chonnam National University, Gwangju, Republic of Korea; ^2^ Department of Bioenergy Science and Technology, Chonnam National University, Gwangju, Republic of Korea; ^3^ Bio-Energy Research Center, Chonnam National University, Gwangju, Republic of Korea; ^4^ Kumho Life Science Laboratory, Chonnam National University, Gwangju, Republic of Korea

**Keywords:** ER body, NAI2, EFE domain, NAI2 domain, homomeric interaction, ER body formation

## Abstract

The endoplasmic reticulum (ER) serves essential functions in eukaryotic cells, including protein folding, transport of secretory proteins, and lipid synthesis. The ER is a highly dynamic organelle that generates various types of compartments. Among them, the ER body is specifically present in plants in the *Brassicaceae* family and plays a crucial role in chemical defense against pathogens. The NAI2 protein is essential for ER body formation, and its ectopic overexpression is sufficient to induce ER body formation even in the leaves of *Nicotiana benthamiana*, where the ER body does not naturally exist. Despite the significance of NAI2 in ER body formation, the mechanism whereby NAI2 mediates ER body formation is not fully clear. This study aimed to investigate how two domains of *Arabidopsis* NAI2, the Glu-Phe-Glu (EFE) domain (ED) and the NAI2 domain (ND), contribute to ER body formation in *N. benthamiana* leaves. Using co-immunoprecipitation and bimolecular fluorescence complementation assays, we found that the ND is critical for homomeric interaction of NAI2 and ER body formation. Moreover, deletion of ED induced the formation of enlarged ER bodies, suggesting that ED plays a regulatory role during ER body formation. Our results indicate that the two domains of NAI2 cooperate to induce ER body formation in a balanced manner.

## Introduction

The endoplasmic reticulum (ER) serves essential functions in eukaryotic cells, including synthesis of secretory proteins and those localized at endomembrane compartments. In addition, the ER is responsible for various mechanisms of posttranslational modifications, such as glycosylation and disulfide-bond formation, to ensure proper folding and functionality of proteins. Moreover, the ER possesses mechanisms known as ER-associated degradation, whereby misfolded proteins are retrotranslocated from the ER and subsequently degraded by the proteasome (Strasser, 2018; Chen et al., 2020; Wang and Wang, 2023). Physiological responses of plants to environmental changes are highly correlated with all of these events occurring in the ER (Liu et al., 2011; Ahn et al., 2018; Reyes-Impellizzeri and Moreno, 2021).

Plants have several types of ER-derived compartments, such as protein bodies, lipid droplets, and the ER body, which carry out specialized functions for normal plant growth (Nakano et al., 2014; Brocca et al., 2021; Rufian et al., 2021; Choi et al., 2022; Li et al., 2022). Among them, the ER body is specifically found in *Brassicaceae* plants, which include *Arabidopsis thaliana*. It has a spindle-shaped structure, with an approximate diameter of 1 μm and length of 10 μm (Matsushima et al., 2003a). In *Arabidopsis*, the ER body is constitutively present in the seedlings and roots, whereas in rosette leaves, its formation is induced by wounding or treatment with jasmonic acid (Matsushima et al., 2002; Ogasawara et al., 2009; Gotte et al., 2015). The major constituents of the ER body are β-glucosidases, including PYK10/BGLU23 (Matsushima et al., 2003b; Nakano et al., 2014). These β-glucosidases are involved in chemical defense against herbivores and pathogens by providing defensive compounds from the glucosinolates in the vacuoles (Nakazaki et al., 2019; Yamada et al., 2020; Lv et al., 2022). The basic helix-loop-helix transcription factor NAI1 is considered essential for constitutive ER body formation, since loss-of-function mutations of *NAI1* impair ER body formation (Matsushima et al., 2004). Moreover, NAI1 transcriptionally upregulates *PYK10/BGLU23*, *NAI2*, *membrane of ER body1* (*MEB1*), and *MEB2*, all of which encode pivotal components in ER body formation (Matsushima et al., 2004; Yamada et al., 2013; Sarkar et al., 2021). NAI2 is specifically localized to the ER body and functions as a main determinant for ER body formation. In the absence of NAI2, PYK10 and MEB1/2 diffuse in the lumen and membrane, respectively, of ER network, instead of being localized to the ER body (Yamada et al., 2008; Yamada et al., 2013). Moreover, ectopic overexpression of *Arabidopsis* NAI2 in the leaves of *Nicotiana benthamiana*, which does not belong to *Brassicaceae* plants, is sufficient to strongly induce ER body formation (Geem et al., 2019). All these observations indicate that NAI2 is a determining factor in ER body formation.

Previously, Geem et al. (2019) have shown that TSA1, a homolog of NAI2, is involved in ER body formation induced by wounding stress or treatment with jasmonic acid. In addition, TSA1 and NAI2 form a heteromeric complex, and these two proteins show an additive effect on ER body formation in the leaves of *N. benthamiana* (Geem et al., 2019). NAI2 contains the N-terminal signal sequence required for ER targeting, 10 repeats of Glu-Phe-Glu (EFE) motif, which we named EFE domain (ED) in this study, and a C-terminal NAI2 domain (ND) (Yamada et al., 2008). Considering that NAI2 and TSA1 have similar domain organization, the interaction between the two proteins suggests the possibility of homomeric interaction of NAI2.

Accordingly, this study aimed to elucidate the specific roles of the ED and ND of *Arabidopsis* NAI2 in the homomeric interaction of NAI2 and ER body formation. Co-immunoprecipitation and bimolecular fluorescence complementation (BiFC) assay revealed that the ND of NAI2 is critical for the homomeric interaction. Furthermore, the overexpression of an NAI2 mutant in which the ED was deleted, enhanced the ER body formation. All these results suggest that the ND of NAI2 is critical for ER body formation and that the ED plays a regulatory role to ensure balanced ER body formation.

## Material and methods

### Plant materials and growth conditions

*N. benthamiana* plants were grown on soil in a greenhouse at 23–24 °C and with 40–65% relative humidity and a 16-h light/8-h dark cycle. The leaves of 6–7-week-old plants were used for agro-infiltration.

### Plasmid DNA construction

The constructs containing the sequence encoding the NAI2ΔED or NAI2ΔND were generated using polymerase chain reaction (PCR)-based mutagenesis (Lee et al., 2006). To generate *NAI2ΔED : HA* or *NAI2ΔED : GFP*, in which the sequence encoding the ED was deleted, the construct *NAI2-HA* (Geem et al., 2019) was used as the template for PCR using the following primer sets: *Xba*I-NAI2-forward (5′-GCTCTAGA ATGGGAACAAAGTTTTTAGC-3′)/NAI2ΔED-reverse (5′-GTTGATGGATTCTTTGCAGATAACTCAGCTGATGTATCAA-3′) and NAI2ΔED-forward (5′-TTGATACATCAGCTGAGTTATCTGCAAAGAATCCATCAAC-3′)/NosT (5′-GAACGATCGGGGAAATTC-3′). Subsequently, the two PCR products were subjected to overlapping PCR using the primers *Xba*I-NAI2-forward and NosT. Afterward, the PCR products were digested using the *Xba*I and *Bam*HI restriction endonucleases, and then the products were ligated to a *GFP* or *HA*-containing pUC-based expression vector that had been digested using the same restriction endonucleases. The resulting constructs were digested using the *Xba*I and *Eco*RI restriction endonucleases, and then the digests were ligated to the pCambia1300 plant-expression vector that had been digested using the same restriction endonucleases. To generate *NAI2ΔND-HA* or *NAI2ΔND-GFP* in which the sequence encoding the ND was deleted, the construct NAI2-HA was used as the template for PCR using the primers *Xba*I-NAI2-forward and *Bam*HI-NAI2ΔND-reverse (5′-CGGGATCC GTTTTCTCCATTAGCCTTTGC-3′). The PCR products were digested using the *Xba*I and *Bam*HI restriction endonucleases, and then the digests were ligated to a *GFP* or *HA*-containing pUC-based expression vector that had been digested using the same restriction endonucleases. The resulting construct was digested using the *Xba*I and *Eco*RI restriction endonucleases, and then the digests were ligated to the pCambia1300 plant-expression vector that had been digested using the same restriction endonucleases. In addition, for bimolecular fluorescence complementation (BiFC) assay, the full-length *NAI2* as well as the *NAI2ΔED* and *NAI2ΔND* fragments generated *via Xba*I/*Bam*HI digestion were ligated to a pCambia1300 binary vector harboring the sequences encoding the N-terminal 173 residue-long fragment (NV) or the C-terminal 83 residue-long fragment (CV) of Venus fluorescent protein that had been digested using the same restriction endonucleases.

### Agro-infiltration of binary constructs into *N. benthamiana* leaves

*Agrobacterium tumefacians* (EHA105) was transformed with the binary vector constructs generated in this study. *A. tumefacians* cells harboring these constructs were introduced into *N. benthamiana* leaves *via* syringe infiltration as described previously (Islam et al., 2019). *A. tumefacians* harboring the *p38* sequence of turnip crinkle virus, which encodes a suppressor of host gene-silencing machinery, was co-infiltrated during each agro-infiltration procedure (Islam et al., 2019).

### Co-immunoprecipitation, western-blot analysis, and blue-native polyacrylamide gel electrophoresis

Leaves (200 mg), harvested 5 d after the agro-infiltration, were ground in liquid nitrogen. Total protein extracts were prepared using 1 ml of IP buffer (50 mM Tris-HCl [pH 7.5], 150 mM NaCl, 0.1% Triton X-100, and 1× protease inhibitor cocktail). After incubation at 4 °C for 15 min, the samples were subjected to centrifugation at 3000 g for 10 min. The resulting supernatants were incubated with an anti-GFP antibody overnight at 4 °C, followed by incubation with protein-A beads for 3 h at 4 °C. The immunoprecipitated samples were washed three times with the IP buffer. Afterward, the total and immunoprecipitated samples were analyzed *via* western blotting with anti-GFP and anti-HA antibodies. In every co-immunoprecipitation experiment, 20 μL of total protein extracts (2% of total volume) were used as total fractions in SDS-PAGE, and all of the remaining samples were subjected to immunoprecipitation with anti-GFP antibody. After that, one-third and two-thirds of immunoprecipitated samples were used for Western blotting with anti-GFP and anti-HA antibodies, respectively. Western blotting (Geem et al., 2021) and BN-PAGE (Kim et al., 2022) were performed as described previously.

### Subcellular fractionation

Transformed leaves (0.4 g) were homogenized in 3 mL of fractionation buffer (50 mM HEPES-NaOH, pH 7.5, 5 mM EDTA, 0.4 M sucrose, and protease inhibitor cocktail (Roche)) on ice using a razor blade. The homogenates were then filtered through miracloth (Calbiochem) to remove debris, followed by centrifugation at 1000 g at 4°C for 20 min. The pellet fraction was resuspended in 500 μL of fractionation buffer and stored as the P1 (ER body) fraction. The supernatant was then subjected to centrifugation at 8000 g at 4°C for 20 min. The supernatant from the second centrifugation was subjected to centrifugation at 100000 g at 4°C for 1 h. After that, the pellet fraction was resuspended in 500 μL of fractionation buffer and stored as the P100 fraction.

### Confocal laser-scanning microscopy

Fluorescent images of the leaves were taken *via* CLSM 5 d after agro-infiltration. The filter sets had an excitation wavelength/spectral detection bandwidth of 488 nm/514 nm for BiFC, 488 nm/500–530 nm for GFP, and 543 nm/560–615 nm for mCherry. We observed 50 GFP-positive cells in each transformation. The fluorescent pattern observed in more than 95% of GFP-positive cells was considered representative of the localization in this study.

## Results and discussion

### The ND is critical for homomeric interaction of NAI2

**Figure 1:**
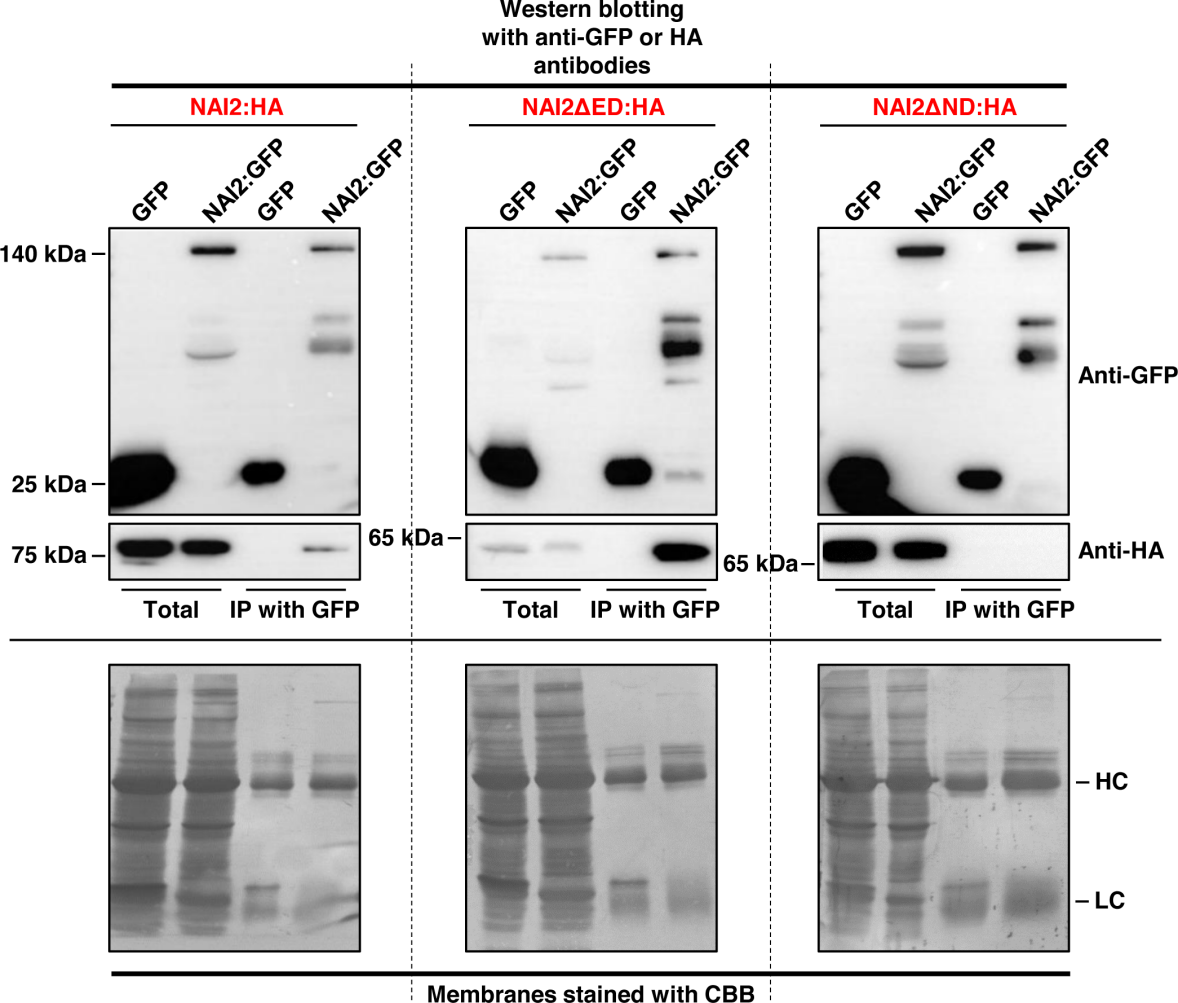
Homomeric interaction of the NAI2 protein is mediated by the NAI2 domain. *N. benthamiana* leaves were transformed with *NAI2:HA*, *NAI2ΔED : HA*, or *NAI2ΔND : HA*, alongside *GFP* or *NAI2:GFP*. After 5 d, the leaves were lysed, and the lysates were subjected to immunoprecipitation using an anti-GFP antibody, followed by western blotting with anti-GFP and anti-HA antibodies. The 2% of total protein extracts was used as ‘Total’ fractions, and one-third and two-thirds of immunoprecipitated samples were used for Western blotting with anti-GFP and anti-HA antibodies, respectively. HC, antibody heavy chain; LC, antibody light chain; CBB, Coomassie brilliant blue.

Previously, Geem et al. (2019) have shown the interaction between NAI2 and TSA1, both of which have similar domain organization (Geem et al., 2019), raising the possibility of homomeric interaction of NAI2. To investigate whether NAI2 undergoes homomeric interaction, we transformed the leaves of *N. benthamiana* with *NAI2:HA* and *GFP*, or with *NAI2:GFP via* agro-infiltration (**Figure 1**). After 5 d, leaf extracts were prepared and then subjected to immunoprecipitation using an anti-GFP antibody, followed by western blotting using an anti-HA antibody. NAI2:HA interacted with NAI2:GFP but not with the negative control (GFP) (**Figure 1**).

**Figure 2:**
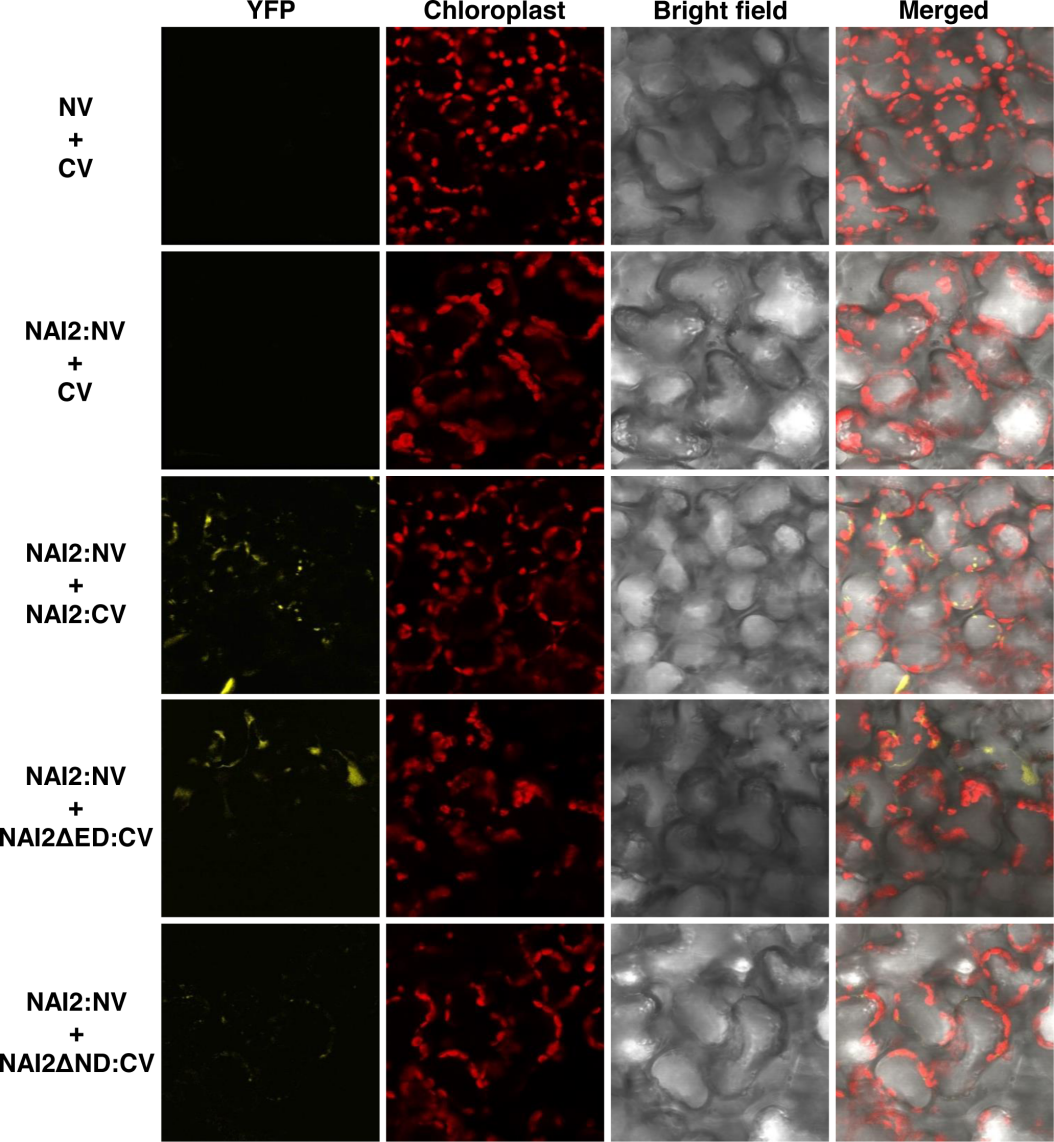
The NAI2 domain is mainly involved in self-assembly of the NAI2 protein. *N. benthamiana* leaves were transformed with *NAI2:NV* alongside *CV*, *NAI2ΔED : CV*, or *NAI2ΔND : CV*. After 5 d, the transformed leaves were examined *via* confocal laser scanning microscopy. Yellow signals: fluorescent signals of Venus fluorescent protein. Red signals: chlorophyll autofluorescence. NV and CV: N- and C-terminal fragments of Venus, respectively.

Next, to identify the domain involved in this interaction, the constructs *NAI2ΔED : HA* and *NAI2ΔND : HA*, which lack the sequences encoding the ED and ND, respectively, were generated. To assess for the interaction between these mutants and NAI2:GFP, we performed co-immunoprecipitation assay (**Figure 1**). We observed that NAI2ΔED : HA, but not NAI2ΔND : HA, interacted with NAI2:GFP, indicating that the ND is involved in NAI2 homomeric interaction (**Figure 1**). Considering the nature of co-immunoprecipitation assay, which shows not only direct but also indirect interactions, we performed BiFC assay to check whether homomeric interaction of NAI2 is mediated through direct interaction (**Figure 2**). NAI2:NV (N-terminal fragment of Venus fluorescent protein) interacted with NAI2:CV (C-terminal fragment of Venus fluorescent protein) or NAI2ΔED : CV but not with CV alone. NAI2:NV showed weak interaction with NAI2ΔND : CV, which is not consistent with the results from the co-immunoprecipitation assay (**Figures 1**, **2**). One possibility is that although ED served a minor role in NAI2 homomeric interaction, its contribution was too weak to be detected by the co-immunoprecipitation assay. In addition, it is also plausible to consider that this discrepancy may have resulted from the nature of the BiFC assay, in which the interaction between NV and NC, mediated by any weak interaction between two proteins, can be irreversible. (Miller et al., 2015). Together, these results indicate that NAI2 undergoes homomeric interaction, and this interaction is mainly mediated by the ND of NAI2.

### The ND is critical for ER body formation, whereas the ED appears to play a regulatory role

**Figure 3:**
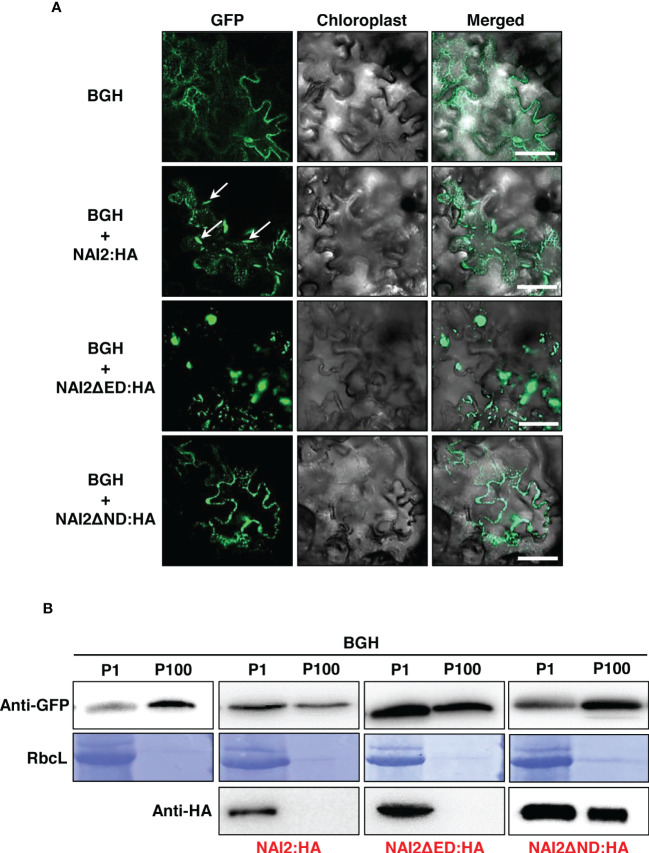
The NAI2 domain of the NAI2 protein is critical for ER body formation. **(A)** Subcellular localization of BiP : GFP:HDEL (BGH). *N. benthamiana* leaves were transformed with *BGH* alongside *NAI2:HA*, *NAI2ΔED : HA*, or *NAI2ΔND : HA*. After 5 d, the transformed leaves were examined *via* confocal laser scanning microscopy. Green signals: fluorescence of GFP. Arrow: ER body. **(B)** Subcellular fractionation. The P1 and P100 fractions of the lysates of the transformed leaves were isolated and then subjected to western blotting with anti-GFP and anti-HA antibodies. RbcL: Rubisco large subunit stained with Coomassie brilliant blue.

Previously, it has been shown that the ER marker protein BGH, in which GFP is N-terminally fused with the signal peptide of BiP and C-terminally fused with BiP’s ER retention signal, localizes to the ER lumen but becomes accumulated in the ER body during ER body formation. (Yamada et al., 2008; Geem et al., 2019). To investigate how the ND and ED contribute to ER body formation, we transformed the leaves of *N. benthamiana* with *BGH* alongside wild-type *NAI2:HA*, *NAI2ΔED : HA*, or *NAI2ΔND : HA*. After 5 d, we examined the GFP-expression patterns *via* CLSM (**Figure 3A**). Consistent with our previous results, overexpression of NAI2:HA was sufficient to induce ER body formation (Geem et al., 2019). However, NAI2ΔND : HA failed to induce the formation of the ER body. Moreover, BGH exhibited punctate staining patterns together with the ER network pattern in the presence of NAI2ΔND : HA, suggesting that NAI2ΔND : HA somehow affected ER morphology (**Figure 3A**). Interestingly, the overexpression of NAI2ΔED resulted in enlarged and rounded ER bodies, suggesting that although the ED is not the main determinant for ER body formation, it somehow plays a regulatory role in ER body formation, thereby enabling balanced ER body formation (**Figure 3A**). Next, to confirm these results at the biochemical level, we performed subcellular fractionation of the lysates of the transformed leaves. As previously described, P1 fraction was found to contain ER body components (**Figure 3B**) (Yamada et al., 2013; Geem et al., 2019). As expected, BGH was mainly present in the ER-network–rich fraction (P100), in the normal condition or co-expression of NAI2ΔND : HA, which lacks the ND (**Figure 3B**). However, when co-expressed with the wild-type (NAI2:HA) or NAI2ΔED : HA mutant construct, BGH was accumulated in the P1 fraction more than in the P100 fraction (**Figure 3B**). These results further confirm the importance of the ND of NAI2 in ER body formation.

**Figure 4:**
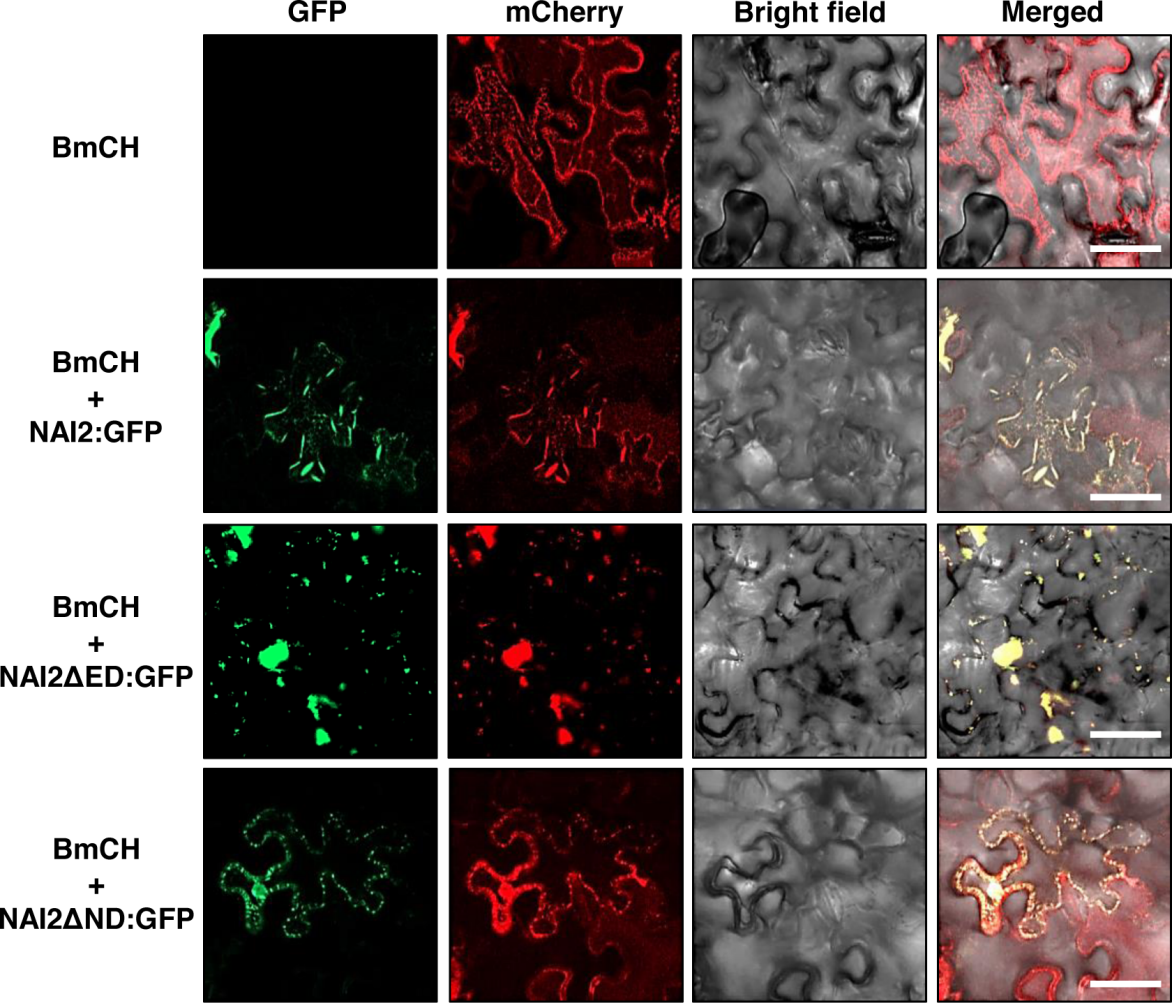
The NAI2 domain of the NAI2 protein is critical for the accumulation of the ER marker protein BiP:mCherry : HDEL in the ER body. Subcellular localization of BiP:mCherry : HDEL (BmCH). *N. benthamiana* leaves were transformed with *BmCH* alongside *NAI2:GFP*, *NAI2ΔED : GFP*, or *NAI2ΔND : GFP*. After 5 d, the transformed leaves were examined *via* confocal laser scanning microscopy. Green signals: fluorescence of GFP. Red signals: fluorescence of mCherry.

Previously, Geem et al. (2019) have shown that overexpression of TSA1:GFP leads to the accumulation of an ER marker protein, BiP:mCherry : HDEL (BmCH), to the ER body. The BmCH construct is a marker protein in which mCherry fluorescent protein is N-terminally fused with the signal peptide of BiP and C-terminally fused with BiP’s ER retention signal. (Geem et al., 2019). To investigate whether GFP-fused wild-type NAI2 or two deletion mutants result in the accumulation of BmCH in the ER body, the leaves of *N. benthamiana* were transformed with *BmCH* alone or together with *NAI2:GFP*, *NAI2ΔED : GFP*, or *NAI2ΔND : GFP* (**Figure 4**). Every BmCH signal was mostly co-localized with GFP-fused wild-type NAI2 or the two deletion mutants (**Figure 4**). Although NAI2:GFP displayed the typical ER body pattern, NAI2ΔED : GFP displayed abnormal ER body patterns, which are remarkably similar to the BGH patterns in the presence of co-expressed NAI2ΔED : HA (**Figures 3A** **,** **4**). However, NAI2ΔND : GFP failed to induce ER body formation.

Taken together, these results suggest that the ND of NAI2 is the major domain involved in the formation of the ER body. In addition, the ED is dispensable for the NAI2-driven ER body formation but may contribute to balanced formation of the ER body.

In this study, we elucidated the specific roles of the ED and ND of NAI2, which is a critical factor for ER body formation. First, we showed that NAI2 undergoes homomeric interaction. Previously, it has been shown that NAI2 and TSA1 form a heteromeric complex, and overexpression of these two proteins induces ER body formation in an additive manner (Geem et al., 2019). Given that the homomeric interaction of NAI2 is mediated by the ND (**Figures 1** and **2**), it is plausible to consider that the heteromeric interaction between NAI2 and TSA1 is also mediated by the NDs of the two proteins. Intriguingly, the overexpression of NAI2ΔED : HA induced the formation of enlarged and rounded ER bodies (**Figure 3**). However, the NAI2ΔND : HA mutant, which does not undergo a robust homomeric interaction, failed to induce ER body formation. Thus, it is possible that the oligomerization of NAI2 or TSA1 is a prerequisite for inducing ER body formation. In fact, there is an additional homologue of *NAI2*, which is *At3g15960* (Yamada et al., 2008). Interestingly, the protein encoded by *At3g15960* lacks the ED. Thus, in the future, it will be necessary to elucidate the spatiotemporal expression pattern of At3g15960 and the roles of this protein in gene expression, ER body formation, and the homomeric and heteromeric interactions among NAI2 and TSA1.

**Figure 5:**
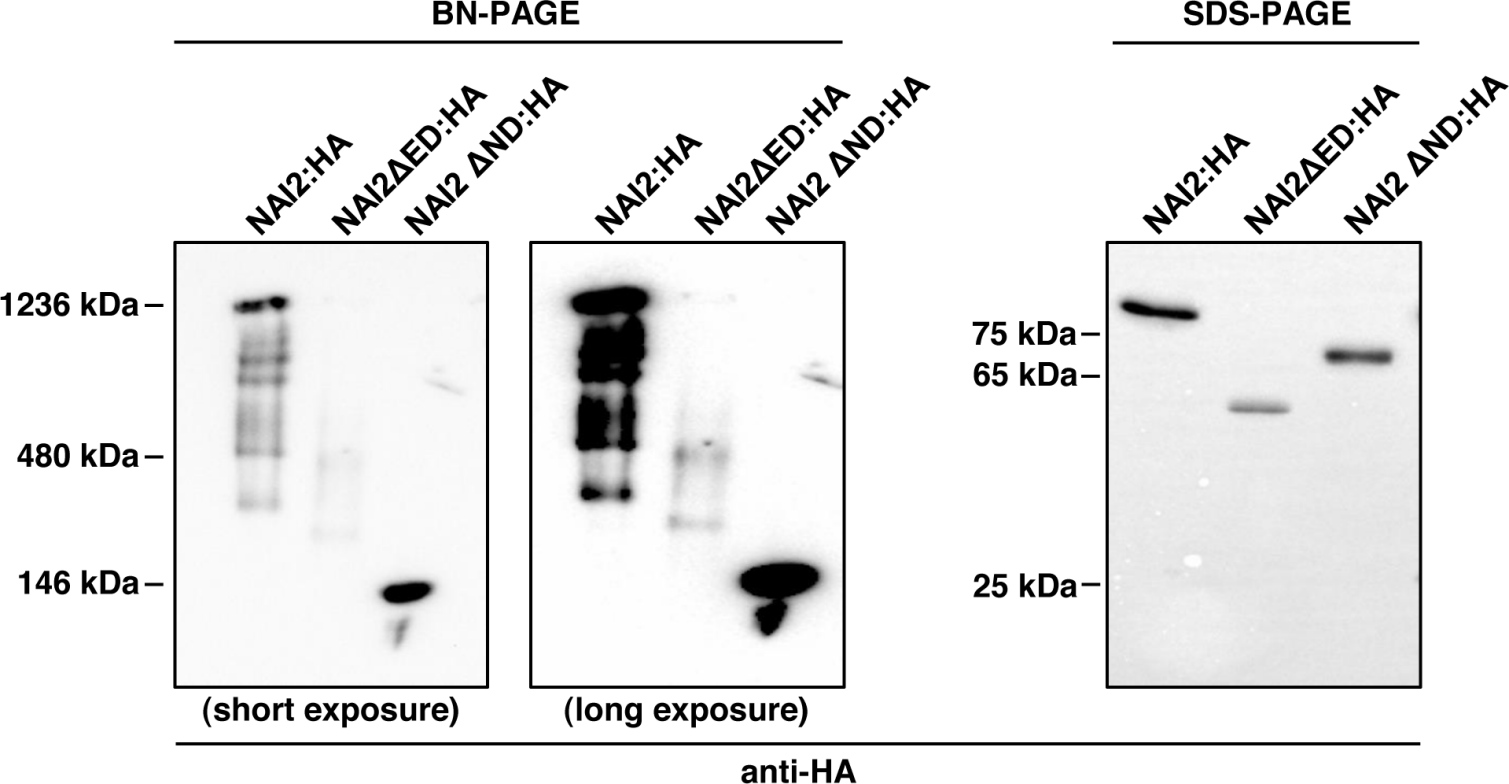
The NAI2 domain is important for the interaction of the NAI2 protein with cellular factors. After 5 d, the lysates of the leaves were subjected to blue-native polyacrylamide gel electrophoresis (PAGE) and sodium dodecyl sulfate-PAGE, followed by western blotting with an anti-HA antibody.

Then what is the role of the ED? The behaviors of BGH and BmCH in the presence of NAI2ΔED : HA (**Figure 3**) and NAI2ΔED : GFP (**Figure 4**), respectively, may give a clue for this question. In the presence of the NAI2 mutants that lack the ED, both ER marker proteins showed fluorescent signals that clearly indicated abnormal ER body patterns. These results suggest that although the ED does not play a determining role in ER body formation, it may be involved in the regulation of ER body formation. During ER body formation, NAI2 may interact with several protein factors, such as MEB1 and MEB2 (Yamada et al., 2013). To gain some insights into how the ED and ND of NAI2 interact with other protein factors, we subjected the lysates of leaves transformed with *NAI2:HA*, *NAI2ΔED : HA*, or *NAI2ΔND : HA* to BN-PAGE (**Figure 5**). Both NAI2:HA and NAI2ΔED : HA formed high-molecular weight complexes > 480 kDa. However, NAI2ΔND : HA produced a band of approximately 140 kDa in BN-PAGE, implicating that the ED alone does not actively interact with other cellular proteins (**Figure 5**). These results suggest that the ND but not the ED of NAI2 may be critical for the interaction with other protein partners, thereby contributing to ER body formation. In the future, it will be necessary to investigate how the ND coordinates ER body formation through interaction with other cellular factors and how the ED ensures balanced ER body formation.

## Data availability statement

The original contributions presented in the study are included in the article/supplementary material. Further inquiries can be directed to the corresponding authors.

## Author contributions

DL and KG conceived this study. YC and KG performed most of the experiments. JK and DL supervised the project and wrote the manuscript. All the authors contributed to the article and approved the submitted version.
